# Towards a sociology of arts and health: how can sociology reveal ‘that which is hidden’?

**DOI:** 10.1177/17579139241290391

**Published:** 2025-07-05

**Authors:** K Warran

**Affiliations:** Public Health, Arts, Theory, Sociology (PATHS) Research Group, School of Health in Social Science, The University of Edinburgh, Edinburgh EH8 9AG, UK

## Abstract

This article explores the potential insights that increased sociological work could bring to the field of arts and health, suggesting that these insights can play a key role in understanding the role of society in the constitution and sustainability of this interdisciplinary field.


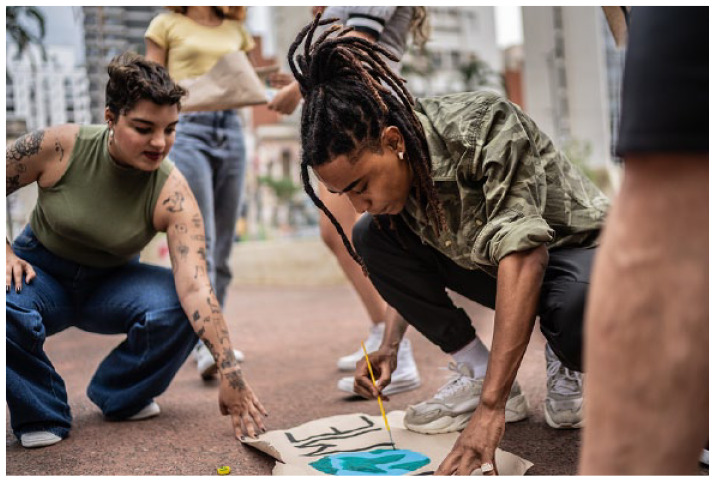


Over the past two to three decades, there has been a rapid increase in research exploring how arts programmes as public health interventions can support the prevention, management, and treatment of a range of health conditions.^1^ However, the application of sociological theories and approaches within research on ‘arts and health’ are at the fringes of the field, with approaches from psychology and public health seeking to evidence the impact of arts interventions at the forefront. There are many reasons why this may be from a political perspective. For example, evidence-based policymaking and funding priorities, such as those set by governments, trusts, foundations, and others, underpin the sociopolitical and economic sustainability of the field, particularly in the West. Yet, given the clear structural elements that contribute to how the field of arts and health is constituted, it is surprising that more sociological perspectives and theories have not yet been brought into the field to further unpack, question, critique, and deepen understanding of the role of society in arts and health activities. The function of sociology, Pierre Bourdieu argued, ‘is to reveal that which is hidden’.^2^ This begs the question: What can sociology reveal about what may be hidden in ‘arts and health’? This short article discusses the potential insights that increased sociological work could bring to the field of arts and health.

In the context of the sociology of the arts, Zolberg^3^ explained that sociologists treat ‘art’ as something to be deconstructed to reveal aspects of social structure and process – something that embodies these ‘hidden’ aspects. She argues that art can be viewed as a synecdoche: representative of a total social experience.^3^ There are many ways one could approach exploring the ‘total social experience’ of arts and health. To just name a few, this could include exploring power struggles between institutions and people, affected by class and acquisition of capital,^4^ ‘patterns of collectivity’ between and across cooperative networks of stakeholders (e.g. artists, healthcare professionals, participants),^5^ or flows of affects within arts-health assemblages of ‘bodies, things, ideas and institutions’.^6,7^ Other avenues include analysing the expression of certain shared beliefs and values (e.g. the importance of the role of the arts in health) as part of close-knit social groups with a shared sense of morality,^8^ applying an ecosocial lens to consider arts engagement as determined by interconnected factors across multiple social levels,^9^ or viewing the field of arts and health itself as a social movement, with those in the field acting as boundary workers to maintain epistemological divisions.^10^ All of these social experiences take place within a particular moment in time, history, and politics. As has been argued by Williams,^11^ the agenda of arts and health aligns with a ‘regressive neoliberal agenda’ and, as such, the political and economic context of its existence explains the dominant focus on evidence-based research from those working in the field.^12^

Yet, while critical perspectives have started to be introduced into the field of arts and health in recent years, the inclusion of sociological approaches within this has been minimal, and many of these perspectives have tended to uphold a medical hierarchy of evidence of what counts as ‘rigour’, rather than seeking to explore how society has constructed what counts as knowledge and why. Within this, there is sparse acknowledgement that Western notions of ‘knowledge’, ‘arts’, and ‘health’ dominate, with little attention given to decolonising methodologies and methods. Furthermore, while sociology often entails critique and has been described as ‘a meddlesome and often irritating stranger’,^13^ sociology is far from being only about critique. The detailed micro-sociological analysis of music and health from Tia DeNora and colleagues makes this palpable. DeNora explores ‘what actors do’, how they interact with objects, and how they ‘make do with circumstances that exceed their control’, constructing an ‘in action’ perspective.^14^ By focusing on micro-actions, this research elucidates the role of broader aspects of society in how these micro-interactions are constituted. In a study with DeNora and Ansdell,^15^ this perspective is utilised to explore music’s role in processes of change: ‘people engage with it [music] and draw it into interactive webs of significance and practice’. As such, this intricate analysis of the relations and things involved in community music practice leaves the reader with a deeper understanding of what people and music can achieve together.^15^

Even if a researcher were to apply sociological approaches in ways that draw on what has been described as its ‘meddlesome’ side, perhaps this could be important to ensure that the arts and health field can work towards equity. Returning to Bourdieu,^2^ he argued that sociology needs to reveal that which is hidden in order to ‘minimize the symbolic violence within social relations’. Arts and health activities are socially situated, and primarily constructed within the Western world as part of public health strategies. These activities are subject to the same broader social forces of institutional power and inequalities as any other phenomena within late-stage capitalism. If there is any level of ‘unconscious’ or ‘tacit complicity’^2^ in relation to symbolic violence within the field of arts and health, is it not the role of researchers to unpack this further and work towards a more equal society?

Both ‘art’ and ‘health’ have been analysed sociologically independently (as ‘the sociology of art’ and ‘the sociology of health’), exploring how these phenomena are shaped by society and may reveal facets of social structure. Yet, ‘arts and health’ is rarely studied in view of the social forces within which it has been constructed. As an interdisciplinary and intersectoral world that is tightly interwoven with cultural and health policy, healthcare systems, arts institutions, funding bodies, commissioners, community organisations, different arts and cultural groups, and higher educational institutions, bringing together a range of (at times, conflicting) priorities, sociological analyses can play a key role in understanding the social processes of this interdisciplinary field. Such an approach could transform our understanding of the role of the arts in public health, working towards a Sociology of Arts and Health.
